# Conceptual framework for preterm birth review in San Francisco

**DOI:** 10.3389/fpubh.2024.1332972

**Published:** 2024-05-01

**Authors:** Jodi D. Stookey, Sylvia Guendelman, Brady McCallister, Paige Whittemore, Deena Abu-Amara, Maria A. Elsasser, Fardowsa Dahir, Aline Armstrong, Rebecca Jackson

**Affiliations:** ^1^Maternal, Child, and Adolescent Health Division, San Francisco Department of Public Health, San Francisco, CA, United States; ^2^Center of Excellence in Maternal, Child, and Adolescent Health, School of Public Health, University of California, Berkeley, Berkeley, CA, United States; ^3^Dartmouth College, Hanover, NH, United States; ^4^School of Community Health Sciences, University of Nevada, Reno, NV, United States; ^5^School of Nursing and Health Professions, University of San Francisco, San Francisco, CA, United States; ^6^Department of Obstetrics, Gynecology and Reproductive Sciences, University of California, San Francisco, San Francisco, CA, United States

**Keywords:** preterm birth, conceptual framework, social determinants of health, public health monitoring, risk factor patterns

## Abstract

Preterm birth persists as a leading cause of infant mortality and morbidity despite decades of intervention effort. Intervention null effects may reflect failure to account for social determinants of health (SDH) or jointly acting risk factors. In some communities, persistent preterm birth trends and disparities have been consistently associated with SDH such as race/ethnicity, zip code, and housing conditions. Health authorities recommend conceptual frameworks for targeted action on SDH and precision public health approaches for preterm birth prevention. We document San Francisco, California’s experience identifying the need, rationale, methods, and pilot work for developing a conceptual framework for preterm birth review (PTBR) in San Francisco. The PTBR conceptual framework is intended to enable essential public health services in San Francisco that prevent a range of preterm birth phenotypes by guiding plans for data collection, hypothesis testing, analytical methods, reports, and intervention strategy. Key elements of the PTBR conceptual framework are described including, 10 domains of SDH, 9 domains at the whole person level, such as lived experience and health behaviors, 8 domains at the within-person level, such as biomarkers and clinical measures, 18 preterm birth phenotypes, and the interconnections between domains. Assumptions for the PTBR conceptual framework were supported by a scoping review of literature on SDH effects on preterm birth, health authority consensus reports, and PTBR pilot data. Researcher and health authority interest in each of the domains warrants the framework to prompt systematic consideration of variables in each proposed domain. PTBR pilot data, illustrated in heatmaps, confirm the feasibility of data collection based on the framework, prevalence of co-occurring risk factors, potential for joint effects on specific preterm birth phenotypes, and opportunity for intervention to block SDH effects on preterm birth. The proposed PTBR conceptual framework has practical implications for specifying (1) population groups at risk, (2) grids or heatmap visualization of risk factors, (3) multi-level analyses, and (4) multi-component intervention design in terms of patterns of co-occurring risk factors. Lessons learned about PTBR data collection logistics, variable choice, and data management will be incorporated into future work to build PTBR infrastructure based on the PTBR conceptual framework.

## 1 Introduction

Preterm birth remains a leading cause of infant morbidity and mortality around the world, despite decades of intervention effort (1). Interventions against preterm birth have focused on risk factors at the individual level, such as cigarette smoking, malnutrition, stress, or allostatic load (2, 3). Intervention planners have hypothesized that an individual’s behavioral and or psychosocial-risk factors cause preterm birth via physiological pathways, involving endocrine activation, infection, inflammation and /or placental bleeding (2, 3). Conceptual frameworks for thinking about preterm birth have not delineated how Social Determinants of Health (SDH), i.e., the conditions where people are born, grow, live, learn, work and play (4), translate into heterogeneous preterm birth etiology and phenotypes (5). Frameworks that incorporate SDH, whether formulated as explicit hypotheses or implicit expectations about risk factors and outcomes, are needed to inform more effective preterm birth interventions (6).

Preterm birth interventions may have null effects for several reasons. Interventions may fail to account for community-level SDH risk factors in the background, such as racial inequities and poverty (7, 8) or a complex interplay between SDH and individual susceptibility and resilience (9–12). A “shotgun strategy” of combining several intervention strategies into one collective preterm birth intervention may limit intervention quality or dose (13). Phenotype-specific effects of intervention may be diluted if all preterm births are considered as one outcome (14).

To guide intervention planners to think through, and account for, complexity related to co-occurring, interacting risk factors and heterogeneous etiologies in intervention design, we developed the Preterm Birth Review (PTBR) conceptual framework. The following paragraphs describe the setting for the framework development, its structure, pilot testing, possible applications, and limitations to consider in the future. The intent is to improve *ways of thinking* about preterm birth (5) and inform precision public health intervention strategies (15).

## 2 Setting and population

In San Francisco, California, public health monitoring data implicate SDH drivers of preterm birth. Each year, community-level risk factors, including area poverty, public housing, and inadequate prenatal care, are associated with significantly higher preterm birth rates (16, 17). Yet, public health monitoring data lack the detail required to target interventions. It is unknown which specific factor(s) mediate SDH effects on preterm birth. Preterm birth disparities have remained stable in San Francisco, over the past decade, despite efforts of programs and services, such as Women Infants and Children (WIC), Presumptive Eligibility Medi-Cal, Nurse Family Partnership (NFP), CalWorks, and Black Infant Health (BIH), to address food insecurity, barriers to health care, lack of employment leave, low income, and social isolation, respectively.

In 2016–2018, several San Francisco organizations launched efforts to move the needle on preterm birth. The San Francisco Mayor’s Our Children Our Families Initiative, five hospital San Francisco Health Improvement Partnership, University of California San Francisco (UCSF) Benioff California Preterm Birth Initiative (PTBI), and California Department of Public Health (CDPH) Perinatal Equity Initiative each prioritized action to prevent preterm birth (18–20). To provide data to inform local efforts, the San Francisco Department of Public Health, Maternal, Child, and Adolescent Health (SFDPH MCAH) division partnered with hospital, academic, and community partners to define the PTBR conceptual framework and use it to expand monitoring infrastructure to capture more detail about preterm birth risk factors and phenotypes.

## 3 Structure of the preterm birth review conceptual framework

**Figure 1:**
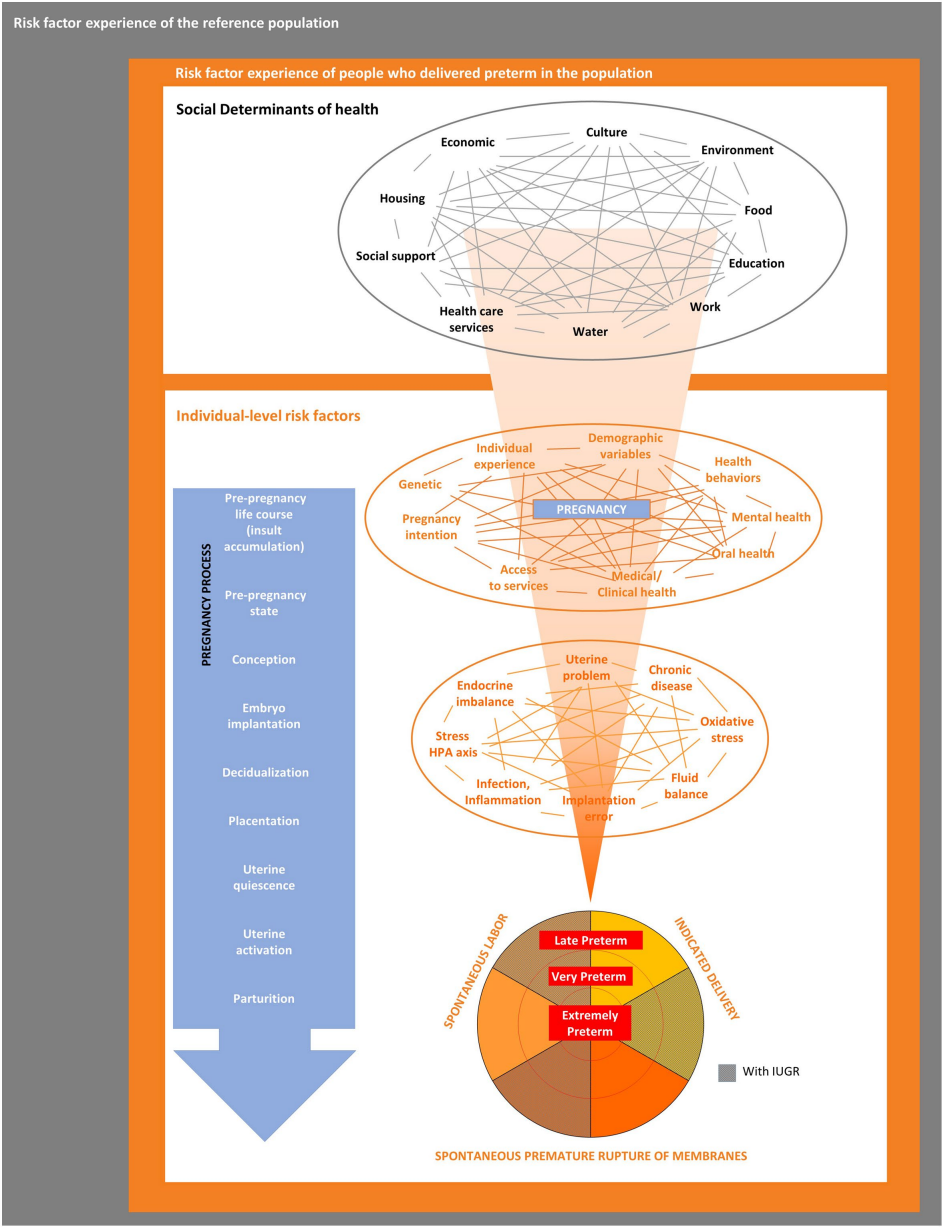
The preterm birth review (PTBR) conceptual framework. The gray box represents the background risk factor experience of the source or reference population. The orange box represents the risk factor experience of people who delivered preterm in the population of interest. Black font represents community-level domains, each representing a type of Social Determinant of Health. Orange font represents individual-level domains, including factors that affect the whole person (e.g., life events and health behaviors) and biomarkers. IUGR, Intrauterine growth restriction.

Figure 1 outlines the PTBR conceptual framework. The key elements of the framework include community-level SDH, individual-level risk factors, and preterm birth phenotype domains, along with interrelationships between levels and domains. The framework posits that SDH act through, or interact with, individual-level risk factors to cause a particular preterm birth phenotype. The framework allows for multiple possible etiologic pathways to preterm birth, potentially happening over a range of critical exposure periods. The framework focuses on risk factors experienced by people who delivered preterm, specifically (the orange box), but defines the risk factors relative to, or in contrast to, the risk factor experience of larger source or specified reference population(s) (represented by the gray backdrop).

### 3.1 SDH domains

At the community level, the PTBR conceptual framework includes 10 SDH domains, each of which may include multiple risk factor measures (see Table 1). The SDH domains were selected following the Dahlgren-Whitehead rainbow model (21), which was familiar to PTBI partners in 2017, though other models are available (6). The Dahlgren-Whitehead model is recognized by the World Health Organization (WHO) for health outcomes, generally (21), and is considered the most widely used model by researchers exploring social health factors (22).

**Table 1:** Number of publications that suggest an association between social determinants of health (SDH)^1^ and preterm birth.

SDH Domain	Search terms	Number of citations retrieved by search terms	Number of citations with SDH risk factor and preterm birth outcome^2^	Examples of variables in each SDH domain that have been studied as possible determinants of preterm birth^3^
Economic	(preterm birth[Title]) AND (poverty[Title/Abstract])	47	28	Income, county, census tract, neighborhood, or zip code poverty, income inequality, index of concentration of extremes, deprivation index, poverty duration, Medicaid
Cultural	(preterm birth[Title]) AND (cultural[Title/Abstract])	27	26	Acculturation, beliefs, social stigma, expectations, norms, attitudes, values, racism, cultural incongruence, transgenerational historical trauma, cultural context, cultural humility, ethnicity, traditions, cultural variation in product use, diet, taboo, residential geographical separation
Environment	(preterm birth[Title]) AND (environment[Title/Abstract])	167	79	Season, ambient temperature, crime, perceived safety, green space, industrial contaminants, air pollution, chemicals, second-hand tobacco smoke, pesticides, nitrates, phthalates, plasticizers, metals, lead
Food	((preterm birth[Title]) AND (food[Title/Abstract]))	47	23	Food insecurity, hunger, food deserts, diet quality, fish, PUFA, added sugar, processed, refined grains, salty snacks, Mediterranean diet, DASH diet, folic acid, iron, calcium, dark green leafy vegetables, fruit, fortification, supplementation, WIC participation, food taboo
Education	(preterm birth[Title]) AND (education[Title/Abstract])	350	236	Maternal education, paternal education, years of schooling, college graduate, health literacy, knowledge transfer, nutrition education
Work	(preterm birth[Title]) AND (employment[Title/Abstract])	38	18	Maternal employment, unemployment, working hours/week, standing, walking, physical workload, job strain, physical demand, lifting, job satisfaction, work in food services, childcare and retail, occupational fatigue, paid maternity leave, guaranteed job protection, regulation of hazardous conditions
Water	(preterm birth[Title]) AND (water[Title/Abstract])	29	13	Community water fluoridation, contaminated drinking water, water quality, nitrates, endocrine disruptor, public water system, running water at home, heat stress, intravenous or oral fluids, dehydration
	(preterm birth[Title]) AND (hydration[Title/Abstract])	23	17	
Health care services	((preterm birth[Title]) AND (prenatal care[Title/Abstract]))	205	106	Preconception, prenatal, or interconception care, family planning, high-risk obstetric care, access to care, quality care, cultural humility in prenatal care
Social support	((preterm birth[Title]) AND (services[Title/Abstract]))	121	62	Medicaid coverage, health visiting or doula services, Black Infant Health program, child protective services, social support, coping mechanisms, behavioral health
	((preterm birth[Title]) AND (social support[Title/Abstract]))	44	33	
Housing	(preterm birth[Title]) AND (housing[Title/Abstract])	20	14	Neighborhood eviction rate, cohabiting, historical redlining, tenure, unstable housing, public housing, homelessness, property conditions, litter, vacancy, residence, mortgage, female-headed household

^1^Search terms were inspired by the Social Determinant of Health (SDH) domains described by Dahlgren and Whitehead (21). The search terms were entered into Pubmed.org to retrieve publications between 1966 and 2022 with no location restrictions applied. English language abstracts and/or full papers retrieved by the search terms were reviewed to identify and count the subset of references that suggest an association between a SDH exposure and preterm birth outcome. Covariates included in analyses as factors to control or account for were considered putative determinants of preterm birth even if not treated as the main exposure variable. ^2^The count excludes studies that report consequences of preterm birth. ^3^The examples, which are not exhaustive, reflect a subset of the topics described in the studies retrieved. References for the examples of variables in each SDH domain are available in the Appendix 1.

Each SDH domain was checked for potential relevance *for preterm birth* risk by scoping review of the PubMed citation database, the most widely used search tool dedicated to biomedical and life sciences literature (23). The scoping review checked for peer-reviewed studies, published in English between 1966 to December 2022, using the search terms listed in Table 1. Search for references in other citation databases, languages, or the bibliographies of each paper was unnecessary because of the large number of references retrieved by PubMed. We counted the number of studies that report an association between each SDH domain, operationalized as exposure, and preterm birth treated as outcome, regardless of the study location or quality. The goal was to cast a broad net for risk factors potentially relevant for pregnant people from all over the world, who deliver in San Francisco, and possible or hypothesized risk factors for preterm birth. Many more than one citation in each of the 10 SDH domains unequivocally indicated researcher interest in preterm birth risk factors each domain, warranting inclusion of all 10 SDH domains in the PTBR conceptual framework. Table 1 includes examples of SDH variables potentially associated with preterm birth reported in the literature.

The PTBR conceptual framework assumes that SDH may be intercorrelated (shown by gray lines in Figure 1). Supporting this assumption, published literature indicates that SDH cluster together and matter in aggregate (24). Opportunities for community residents to obtain financial resources, for example, may depend on redlining policies, types of employment, availability of banks, lending practices, goods and services, costs of living, and taxes (25). Race, low income, food insecurity and inability to pay for housing cluster together (26).

### 3.2 Individual-level domains

At the individual level, the PTBR conceptual framework includes 9 domains that pertain to the whole-person and 8 domains that reflect within-person, biological pathways to preterm birth (see Table 2). All individual-level domains were chosen based on the National Academies of Medicine (NAM) consensus report Preterm Birth: Causes, Consequences, and Prevention (5). Though over a decade old, the consensus report (5) was developed by a committee of experts and highlights risk factors well-recognized by clinicians, which may, today, be routinely documented and available in medical records.

**Table 2:** Individual-level domains in the PTBR conceptual framework with example risk factors.

Conceptual framework domain	Example preterm birth risk factors identified by the National Academy of Medicine
**Whole-person**	
Demographic	Maternal age, marital status and cohabitation, race and ethnicity, individual income, years of education, occupational status (p. 125–133)
Health behaviors	Diet, substance use, sleep, physical inactivity, employment, sexual activity (p. 92–99)
Mental health	Anxiety, depression, trauma, racism, social support, self-esteem, resilience (p. 118–120)
Oral health	Periodontitis, dental care during pregnancy (p. 279–280)
Medical/Clinical health	Chronic hypertension, preeclampsia, pre-pregnancy diabetes, gestational diabetes, anemia, systemic lupus erythematosus, underweight, obesity, asthma, cardiac disease, infection, infertility, assisted reproductive technology, nulliparity, multiple gestations, previous preterm birth, short interpregnancy interval, short cervix (p. 148–154, 265–269, 625)
Access to services	Access to and use of prenatal care, prenatal care adequacy or quality (p. 131, 265, 626)
Pregnancy intention	Unintended, unwanted, or mistimed pregnancy (p. 120)
Genetic	Race, gene–environment interaction, epigenetics, ancestry, family history (p. 207–228)
Individual experience	Divorce, death in the family, illness, injury, job loss, homelessness, chronic and catastrophic stress exposures, life course, experience before pregnancy (p. 37, 104–107)
**Within-person biological pathways to preterm birth**	
Chronic disease	Renal disorders, cardiac disease, hypertension, diabetes, asthma, fetal stress (p. 150)
Oxidative stress, ischemia	Reactive oxygen species, superoxide, nitric oxide, hemorrhage, oxidative damage, thrombin, hypoperfusion (p. 169, 171–175, 191, 294)
Altered fluid balance	Polyhydramnios, oligohydramnios, amniotic fluid composition (p. 178, 181, 191, 209)
Implantation errors	Subfecundity, irregular menses, first trimester vaginal bleeding (p. 16, 60, 158, 187–188)
Infection, inflammation	Bacterial vaginosis, chorioamnionitis, genital tract infection, proinflammatory cytokines (e.g., TNF-alpha), prostaglandin cascade, collagenases (e.g., MMP-1) (p. 169, 176–190)
Stress, HPA axis	Glucocorticoids, corticotropin-releasing hormone (CRH), cortisol (p. 172–180)
Endocrine imbalance	Progesterone, estrogen, oxytocin, epinephrine, angiotensin II (p. 179, 280)
Uterine problems	Tumors, uterine stretch, uterine overdistension, uterine contractility (p. 158, 170, 177, 181)

All of the individual-level domains in the PTBR conceptual framework and example risk factors listed in Table 2 were described by the Institute of Medicine (US) (5).

Whole-person domains in the PTBR conceptual framework include risk factors, such as beliefs, behaviors, or history. Within-person domains in the framework represent physiological systems or biological pathways that can cause preterm birth. Causal pathways to preterm birth are assumed to not be mutually exclusive and may interact or share common downstream cellular and molecular effectors (5). Each individual-level domain may include multiple risk factors, positive resilience factors and/or negative markers of increased risk. Domains that appear at both the SDH and individual levels of the framework, such as education, work, and income, allow the framework to account for resources in the community, such as colleges, workplace policy, and area poverty, as well as individual experiences, such as years in school, time spent working, and individual income.

The PTBR conceptual framework assumes that each SDH measure or pattern may be a potential upstream determinant of one or more individual-level risk factor(s) (11). SDH measures may have interdependent effects on one or more individual-level risk factor(s) (3). Individual-level risk factors may mediate or modify effects of SDH on preterm birth outcomes. An individual’s allostatic load may magnify adverse effects of SDH, while resilience factors may limit adverse effects of SDH on preterm birth (3, 5, 10). Individual-level risk factors can interact (represented by orange lines in Figure 1) to jointly determine preterm birth risk.

### 3.3 Preterm birth phenotypes

The PTBR conceptual framework includes 18 possible preterm birth outcomes, ascertainable from data available on birth records, and defined by three ultimate cause categories, three gestational age periods, and normal- vs. small-for-gestational-age (SGA) growth. The ultimate cause categories were described by the NAM consensus report (5): premature rupture of membranes (PROM), medically indicated delivery, and premature labor. The gestational age periods, which implicate different causal paths within ultimate cause categories (27), follow American College of Obstetrics and Gynecology (ACOG) cutoffs for delivery at <28 weeks, 28 to 33 weeks, or 34 to 36 weeks gestation (28), with gestational age estimated by ultrasound. Birthweight below the 10th percentile for birthweight, based on sex- and gestational age-specific growth curves developed by Olsen et al. (29), was distinguished from birthweight above this cutoff, because fetal growth restriction may suggest causal mechanisms that limit delivery of oxygen or nutrients to the developing fetus (5).

The 18 possible phenotypes allow for common or overlapping risk factors while also leaving room for heterogeneous causal mechanisms that may require different intervention strategy, at different times, before or during pregnancy. PROM occurring before 28 weeks of gestation, if associated with IUGR, for example, may reflect poor placentation (30). In contrast, PROM, occurring before 28 weeks of gestation, without IUGR, might reflect infection (31).

### 3.4 Time

The PTBR conceptual framework assumes that the co-occurring SDH and individual-level risk factor combinations may impact one or more process(es) of pregnancy at one or more stage(s) of pregnancy. The timing of risk factors has implications for when to target interventions. Each individual factor might occur over the life course before pregnancy, immediately before pregnancy, at conception, during embryo implantation, decidualization, placentation, uterine quiescence, uterine activation and/or parturition (5). Risk factor effects may be modified by their timing (3).

## 4 PTBR pilot

Based on the PTBR conceptual framework, we developed a survey, including 77 questions about SDH before and during pregnancy, and a protocol for abstracting 132 elements from the medical record. The SDH questions were selected to align with indicators monitored by SFDPH (16, 17), such as variables from the CDPH Maternal Infant and Health Assessment Survey (32), which are validated and/or standardized for use statewide, so that PTBR results pertaining to people who experienced a preterm birth might be compared with citywide or statewide data. Interrater agreement was checked for information drawn from the medical record about medical history, experience and health/risk behaviors during pregnancy, clinical measures, diagnoses, services and medications during pregnancy, details about the preterm delivery and infant outcomes.

PTBR pilot work is described in more detail elsewhere (33, 34). Briefly, PTBR participants were recruited from the labor and delivery active patient list at one hospital between September 2017 and March 2019. With Labor and Delivery nurse permission, eligible people were approached in hospital to explain the PTBR project, invite participation and obtain informed consent. Patients were offered a $50 gift card for completing the PTBR interview. Permission to approach was denied if patients were distressed or had psychological issues. People younger than 18 years of age and people who did not speak English or Spanish were excluded from PTBR participation. All aspects of the PTBR pilot were approved by the UCSF Institutional Review Board (#17-21932). Survey and medical record data described here were collected from 37 women who delivered a live singleton preterm birth.

### 4.1 Methods for testing conceptual framework assumptions

To check the framework assumption that risk factors co-occur in people who deliver preterm, each risk factor was cross-tabulated with every other risk factor. Using SAS 9.4 software (Cary, North Carolina), every risk factor was expressed as a 0/1 indicator variable. The result from each crosstabulation, the proportion of PTBR participants who reported both factors, was appended to the results from all other crosstabulations to create a dataset containing row factor, column factor, and proportion of participants experiencing both factors as cell values. Risk factors were classified as SDH or individual level factors, and protective, neutral, or adverse factors.

**Figure 2:**
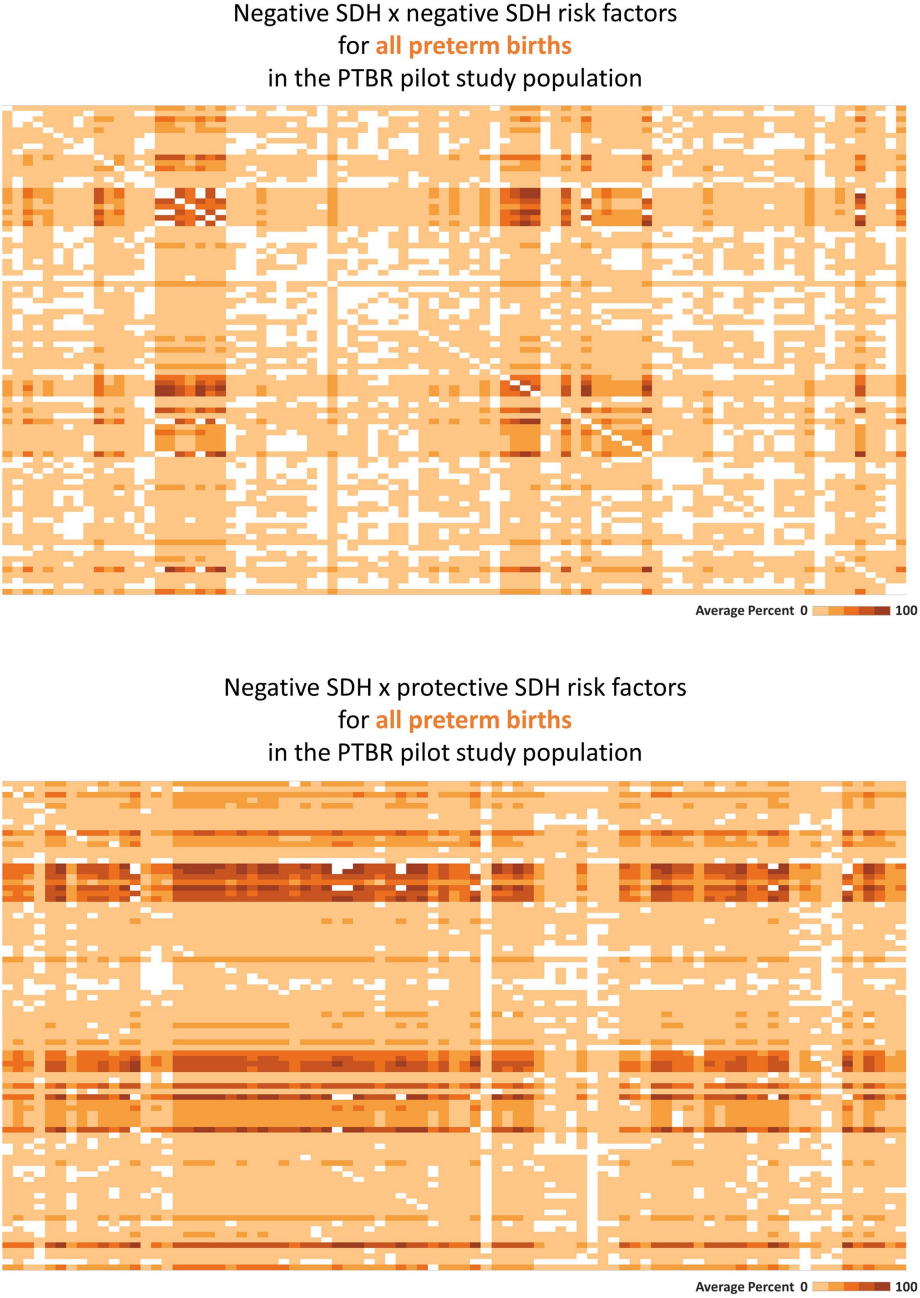
Heatmaps describing co-occurring social determinants of health (SDH). This figure summarizes intercorrelations between SDH risk factors, as hypothesized in Figure 1 (top-most gray circle). Each square on the heatmaps in the figure represents the proportion of the Preterm Birth Review (PTBR) pilot study population who reported both factors in a given combination of SDH risk factors. The color of each square ranges from white to dark orange, representing 0% to 100% of the study population reporting both factors. Labels visible when hovering over each square are not shown on the printed figure. **(A)** The top figure summarizes the co-occurrence of negative SDH assessed, i.e., factors associated with increased risk of preterm birth in the literature. Each row represents one negative social determinant of health (SDH). Each column represents the same list of negative SDH. **(B)** The bottom figure summarizes the co-occurrence of negative SDH with protective SDH that might offset adverse effects of the negative SDH.

**Figure 3:**
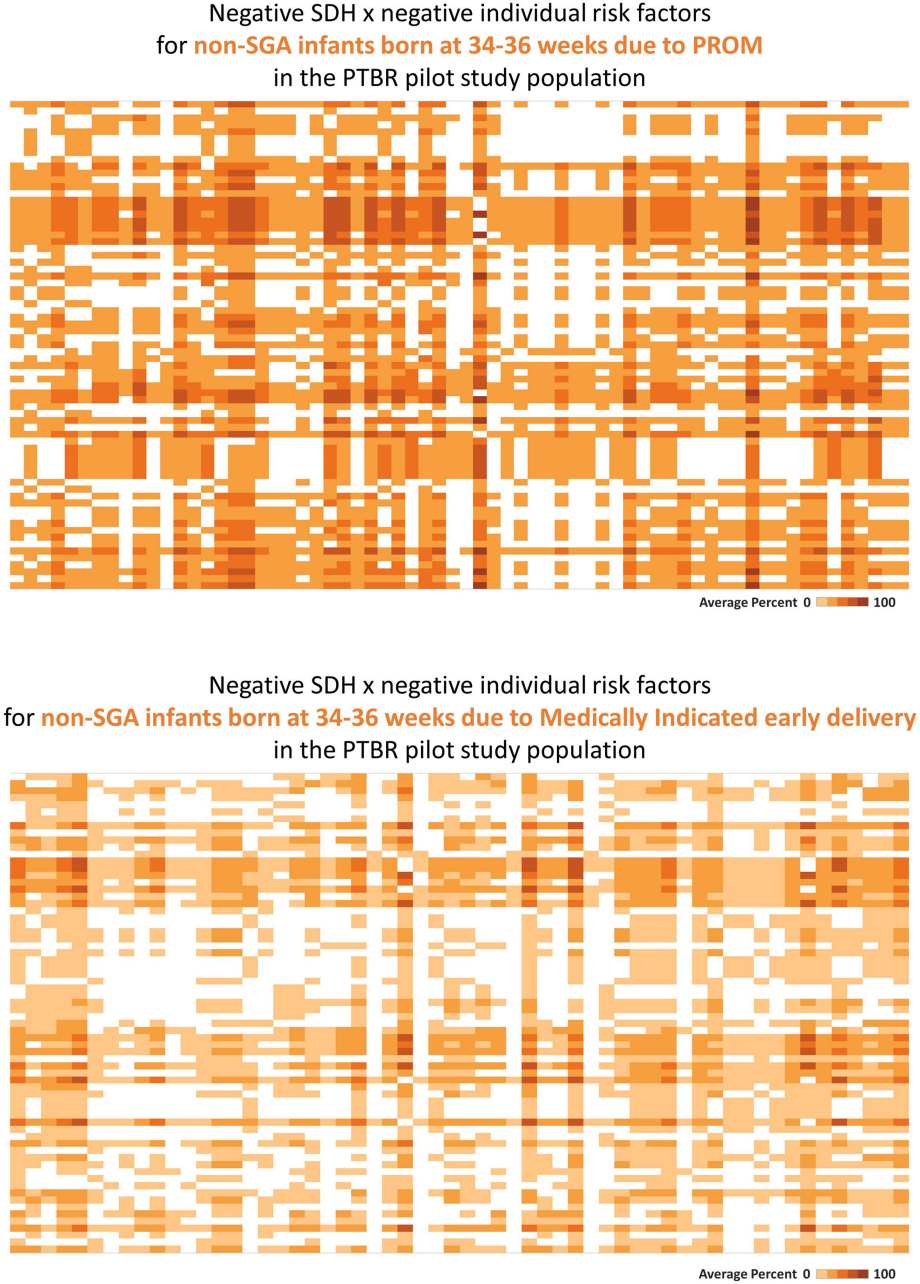
Heatmaps describing phenotype-specific co-occurring social determinants of health (SDH) and individual-level risk factors. This figure summarizes intercorrelations between negative SDH risk factors and individual risk factors which were observed for people with two different preterm birth outcomes. Labels visible when hovering over each square are not shown on the printed figure. **(A)** The top heatmap describes the pattern of risk factors observed for people who delivered a non-small for gestational age (SGA) infant at 34–36 weeks because of premature rupture of membranes (PROM). **(B)** The bottom heatmap describes the pattern of risk factors observed for people who delivered a non-SGA infant at 34–36 weeks because of a medical indication. Each square on the heatmaps in the figure represents the proportion of the group who reported both factors in a given combination of SDH × individual risk factors. The color of each square ranges from white to dark orange, representing 0% to 100% of the group reporting both factors.

Tableau software (Seattle, Washington) was used to visualize selected SDH × SDH (Figure 2) and SDH × Individual factor (Figure 3) crosstabulations as heatmaps. Each square in each heatmap represents the proportion of PTBR participants who experienced a given combination of factors. The square color varies from white to dark orange, representing the range in proportion of PTBR participants who experienced both factors from 0% to 100%, such that a completely white heatmap means that none of the participants experienced any of the combinations of factors. This paper checked heatmaps describing patterns of co-occurring SDH x SDH, adverse SDH x protective SDH, and SDH x individual risk factors to check framework assumptions, only. Comparison of risk factor patterns by phenotype and group is beyond the scope of this paper and will be reported separately.

### 4.2 Pilot results

Consistent with the framework assumption that multiple SDH factors may act together, many SDH risk factors co-occurred in the PTBR sample (see Figure 2, top). Economic hardship co-occurred with issues related to education, food, health service, social service, access to drinking water, safety, and dissatisfaction with the wait-time for prenatal care for participants, for example. White squares in the heatmap pattern signaled that some combinations of SDH did not occur in this study population (i.e., would be irrelevant for local intervention). Adverse SDH did co-occur with many protective SDH factors, including trust in medical providers, daily help at home, feelings of strong community, and social worker support (Figure 2, bottom).

Consistent with assumption that individual-level factors may mediate effects of SDH on preterm birth, SDH factors co-occurred with individual factors (see Figure 3). Premature rupture of membranes following urinary tract infection, for example, co-occurred with clinical measures of dehydration (serum hypernatremia, serum hypertonicity, or elevated urine specific gravity), a recognized cause of urinary tract infection (35), as well as individual behavior (<1 L/d drinking water), and SDH factors that both increase sodium intake [ready-to-eat food (36)] and limit drinking water (no private bathroom, no regular place to sleep, avoidance of public water fountains).

Consistent with assumption that different preterm birth phenotypes may be caused by different patterns of co-occurring SDH and individual risk factors, distinct phenotype specific patterns were observed in the PTBR pilot. In the example in Figure 3, the risk factor pattern for non-SGA infants born at 34–36 weeks because of PROM differed from the risk factor pattern for non-SGA infants born at 34–36 weeks because of medically indicated early delivery. While the risk factor pattern involving PROM included living below the federal poverty line, not having a medical home before pregnancy, missing first trimester prenatal care, not having a regular place to sleep, not having a full kitchen or private bathroom, frequent ready-to-eat meals, dehydration, urinary tract infection, uncontrolled hypertension, smoking, dental problems, and obesity, the risk factor pattern for the Medically indicated early delivery included living below the federal poverty line, subsidized housing, missing prenatal care due to fear of losing the job, worry about bills, and standing at work (variable labels are not shown).

Consistent with assumption that risk factors may interact, PTBR pilot data signaled potential effect modification of SDH effects by other SDH. Income effects, for example, varied by housing and work. Income below the federal poverty line was associated with obesity and dissatisfaction with nutrition support, clinician respect, and social work services for people who did not have a regular place to sleep during pregnancy. For people who were housed and working during pregnancy, in contrast, income below the federal poverty line was associated with depression and substance use.

## 5 Discussion

This paper documents results of a scoping review of peer reviewed literature and a PTBR pilot study. Collectively, the results constitute the PTBR conceptual framework evidence base. The results confirm framework assumptions, set based on reports in the literature about co-occurring SDH risk factors for preterm birth (24–26), individual-level factors mediating SDH effects on preterm birth (3, 11), expert consensus that different preterm birth phenotypes may reflect different risk factors (5), and complex joint or interactive effects (2, 3, 10). The results confirm relevance of the conceptual framework for real-life conditions in San Francisco. Based on the scoping review and pilot results, SFDPH-MCAH and partners will proceed to use the PTBR conceptual framework to develop data collection and reporting infrastructure to expand preterm birth monitoring in San Francisco.

### 5.1 Implications for intervention planners

The PTBR framework is designed to guide planners and researchers to systematically, for each project, consider and define the population group, level of analysis, comparison of interest, and timing. The PTBR conceptual framework focuses attention on *people who delivered preterm*. *Certainty* that the risk factors happened to people who delivered preterm better supports causal inference and program planning than *assumption* of co-occurrence of risk factors and preterm birth, which is standard in current public health monitoring. In our experience, intervention ideas drawn from the incorrect assumption that ecologic data (e.g., zip code level data) represent the exposure experience of people who delivered preterm, waste limited program resources, funding, and time, and fail to help people avoid preterm birth.

The framework prompts intervention designs that account for SDH drivers as well as individual-level effect mediators and modifiers and specify phenotype-specific hypotheses. The framework can support tailoring of intervention components to the local pattern of co-occurring risk factors. Furthermore, the framework can remind planners and researchers to consider collecting data in each and all specified domains and/or remain mindful of unobserved domains or variables as potential sources of error.

The PTBR conceptual framework is meant to identify patterns of risk factors that are prevalent as well as patterns that are absent in the group of interest in a given community and/or area. The risk factor patterns convey potential intervention relevance as well as potential reasons for null effect. For instance, the patterns may guide researchers and planners to improve intervention effectiveness by anticipating and accounting for SDH factors in the background. Risk factor patterns may highlight strengths or resources that can be leveraged by interventions. The PTBR framework might make it possible to identify and develop community resources that do not exist (e.g., freely accessible drinking water and restrooms; blood pressure friendly foods accessible for free, nearby), which might make it possible for health workers to address patient need. Public health nurses, midwives, and doulas, who are charged with efficiently connecting community members with a range of risk factors to care and services, cannot prevent preterm birth if the services needed do not exist.

Consistent with expectation that some risk factors may be irrelevant, locally, the blank squares on the heatmaps in Figures 2, 3 indicate that some risk factors recognized in peer reviewed literature did not occur in San Francisco in the PTBR pilot population and period. Needless to underscore, null effects would be expected for interventions against irrelevant factors. The many bright orange squares, on the other hand, indicate that null effects might also be anticipated for any intervention that does not account for multiple other risk factors.

### 5.2 Implications for data analysis and causal inference

The PTBR conceptual framework has implications for data analysis methods. Unlike methods such as cluster analysis or principal components analysis, which summarize co-occurring variables in a way that does not generalize outside the study population, the present framework provides a benchmark that can be used to map risk factor presence or absence and compare that map over time and across populations or region. Assuming a minimum of one variable in each domain, the PTBR conceptual framework implies grids of at least 10 columns x 10 columns to map SDH x SDH patterns, 10 columns x 15 rows to map SDH x individual factor combinations, and 18 columns x 25 rows to map SDH and individual risk factors that co-occur with each of 18 possible preterm birth phenotypes. As illustrated in Figures 2, 3, the framework motivates heatmap visualizations to check for intercorrelated factors.

The PTBR conceptual framework calls for data analysis methods that can summarize and compare many co-occurring variables, such as principal components analysis, orthogonal projections to latent structures discriminant analysis (OPLS-DA), hierarchical models, path analysis, or neural network analysis. It may provide a structure for artificial intelligence decisions about intervention strategy.

Though the healthy birth outcome is not shown in Figure 1, the PTBR model motivates comparison of preterm birth-related risk factor patterns with reference or control conditions. The model informs choice of inclusion/exclusion criteria and data collection for the reference group(s) to evaluate characteristics associated with preterm birth. The PTBR framework seeks to amplify the experience of the minority who delivered preterm, recognizing that key risk factor patterns may be missed if essential services design initiates from mainstream experience.

The framework suggests analyses that describe risk factor patterns or heatmaps for all people who deliver preterm and/or for sub-groups who deliver preterm. Risk factor heatmaps may be compared by phenotype and/or population sub-groups to check for different drivers and causal mechanisms. Comparative analysis of patterns may help avoid interventions that work for some, but not all phenotypes and population groups, which unintentionally *widen* preterm birth disparities. The heatmaps may highlight co-occurring risk factors, which, if left unaddressed, can limit intervention impact. The temporality or direction of SDH effect on individual factors might be explored using lagged risk factor data.

### 5.3 Lessons learned

The PTBR conceptual framework grapples with complexity related to the intercorrelation and interaction between SDH variables at the community- and individual levels, as well as between individual-level biomarkers. The framework provides a blueprint for developing monitoring infrastructure that can collect, analyze, and report more detailed data about preterm birth.

Through pilot work, we learned that new data collection based on the PTBR framework is feasible (33) and suggests intervention strategies, such as behavioral health services, to buffer against adverse SDH effects (34). Lessons learned about recruitment, data collection logistics, and variable choice and accuracy are described elsewhere (33). While time consuming manual medical record abstraction might be automated, PTBR data collection by survey and/or interview requires patient and staff resources. In San Francisco, where about 700 infants are born preterm annually (16, 17) and each preterm birth carries significant risks and costs for the family and society [e.g., (37)], benefits from improving preterm birth monitoring may justify investment. New AI technology suggests opportunity to reduce costs.

#### 5.3.1 Limitations

The PTBR conceptual framework does not capture SDH other than those described by Dahlgren and Whitehead (22). It does not call out any one SDH as primary root cause, *a priori*, though it does not prevent users from prioritizing a single SDH (e.g., racism in the culture domain) as users apply the framework. The framework focuses on exposures up to the point of preterm delivery and excludes care, outcomes or follow-up for the preterm infant and postpartum parent. Measures that are not covered by standard clinical protocol and insurance reimbursement or newly identified since the NAM consensus report (5) may not be captured by this framework. Finally, the PTBR conceptual framework may be missing key factors and may include factors which co-occur with preterm birth but are not on the causal path. Risk factor patterns identified by PTBR monitoring would need to be tested and confirmed in randomized trials or mendelian randomization analyses.

### 5.4 Conclusions and next steps

The PTBR conceptual framework can be a useful tool for planners and researchers to systematically consider co-occurring community- and individual-level risk factors for preterm birth phenotypes, when planning for data collection, analyses and/or reporting. The framework sets the stage to develop public health services that effectively mitigate adverse effects of *patterns* of risk factors on a range of preterm birth outcomes.

In San Francisco, PTBR work was paused during the COVID-19 pandemic but will restart in 2024. Current plans are to integrate outreach to people who deliver preterm, SDH survey administration, and medical record abstraction into SFDPH-MCAH public health nurse and epidemiology team standard work. The PTBR conceptual model will initially be used to build new infrastructure for patient contact and data collection at each of 5 delivery hospitals, data management and analysis code, and online visualization and reporting. The conceptual model will be available as theoretical basis for intervention planning and evaluation. The PTBR model and infrastructure will be quality improved through iterative Plan-Do-Study-Act cycles. PTBR domains and components may evolve as the model is used and new factors are identified.

We expect that routinely available, systematic PTBR monitoring data will enable detection of risk factor patterns to target for intervention, program evaluation, and quality improvement. The framework may be leveraged to understand how root causes of preterm birth disparities, such as racism (38), act through multiple SDH simultaneously.

## Data availability statement

The original contributions presented in the study are included in the article/Supplementary material, further inquiries can be directed to the corresponding author.

## Ethics statement

This study involving humans was approved by University of California San Francisco Institutional Review Board #17-21932. The study was conducted in accordance with the local legislation and institutional requirements. The participants provided their written informed consent to participate in this study.

## Author contributions

JS: Writing – review & editing, Writing – original draft, Visualization, Validation, Supervision, Software, Resources, Project administration, Methodology, Investigation, Funding acquisition, Formal analysis, Data curation, Conceptualization. SG: Writing – review & editing, Methodology. BM: Data curation, Project administration, Software, Writing – review & editing. PW: Writing – review & editing, Software, Project administration, Data curation. DA-A: Data curation, Project administration, Software, Writing – review & editing, Visualization. ME: Data curation, Project administration, Writing – review & editing. FD: Writing – review & editing, Project administration, Data curation. AA: Resources, Supervision, Validation, Writing – review & editing. RJ: Writing – review & editing, Validation, Supervision, Resources, Project administration, Methodology, Funding acquisition.
