# Supplementary material for: Conceptual framework for preterm birth review in San Francisco
Source: Front Public Health. 2024 May 1;12:1332972. doi: 10.3389/fpubh.2024.1332972 (PMC11094341; doi:10.3389/fpubh.2024.1332972)
Supplement: Supplementary file 1 [file Data_Sheet_1.pdf]

Appendix 1 - References for examples in Table 1

Economic

1. Burris HH, Baccarelli AA, Wright RO, Wright RJ. Epigenetics: linking social and environmental exposures to preterm birth. Pediatr Res. (2016) 79(1-2):136-40. doi: 10.1038/pr.2015.191
2. DeFranco EA, Lian M, Muglia LA, Schootman M. Area-level poverty and preterm birth risk: a population-based multilevel analysis. BMC Public Health. (2008) 8:316. doi: 10.1186/1471-2458-8-316
3. Huynh M, Spasojevic J, Li W, Maduro G, Van Wye G, Waterman PD, Krieger N. Spatial social polarization and birth outcomes: preterm birth and infant mortality - New York City, 2010-14. J Public Health. (2018) 46(1):157-166. doi: 10.1177/1403494817701566
4. Stanhope KK, Hogue CR, Suglia SF, Leon JS, Kramer MR. Restrictive sub-federal immigration policy climates and very preterm birth risk among US-born and foreign-born Hispanic mothers in the United States, 2005-2016. Health Place. (2019) 60:102209. doi: 10.1016/j.healthplace.2019.102209
5. Chambers BD, Baer RJ, McLemore MR, Jelliffe-Pawlowski LL. Using index of concentration at the extremes as indicators of structural racism to evaluate the association with preterm birth and infant mortality-California, 2011-2012. J Urban Health. (2019) 96(2):159-170. doi: 10.1007/s11524-018-0272-4
6. Reagan PB, Salsberry PJ. Race and ethnic differences in determinants of preterm birth in the USA: broadening the social context. Soc Sci Med. (2005) 60(10):2217-28. doi: 10.1016/j.socscimed.2004.10.010
7. Gobaud AN, Kramer MR, Stearns ER, Haley DF. Measuring small-area violent crime: a comparison of observed versus model-estimated crime rates and preterm birth. Ann Epidemiol. (2021) 55:27-33. doi: 10.1016/j.annepidem.2020.11.008
8. Hesson AM, Pitts DS, Langen ES. Delivering equity: preterm birth by socioeconomic class before and after coverage of 17-hydroxyprogesterone caproate. J Matern Fetal Neonatal Med. (2022) 35(24):4713-4716. doi: 10.1080/14767058.2020.1863358

Culture

9. Ruiz RJ, Dwivedi AK, Mallawaarachichi I, Balcazar HG, Stowe RP, Ayers KS, Pickler R. Psychological, cultural and neuroendocrine profiles of risk for preterm birth. BMC Pregnancy Childbirth. (2015) 15:204. doi: 10.1186/s12884-015-0640-y
10. Bronstein JM. The cultural construction of preterm birth in the United States. Anthropol Med. (2020) 27(2):234-241. doi: 10.1080/13648470.2019.1688610
11. Kelley M, Rubens CE; GAPPS Review Group. Global report on preterm birth and stillbirth (6 of 7): ethical considerations. BMC Pregnancy Childbirth. (2010) 10 Suppl 1(Suppl 1):S6. doi: 10.1186/1471-2393-10-S1-S6
12. Smith KL, Shipchandler F, Kudumu M, Davies-Balch S, Leonard SA. "Ignored and Invisible": Perspectives from Black women, clinicians, and community-based

organizations for reducing preterm birth. Matern Child Health J. (2022) 26(4):726-735. doi: 10.1007/s10995-021-03367-1
13. Abbott KM, Boyens BMS, Gubbels JAA. Intergenerational talking circles exploring psychosocial stressors for preterm birth and strategies for resilience among American Indian women. J Transcult Nurs. (2022) 33(3):268-277. doi: 10.1177/10436596221081269
14. Harron K, Verfuerden M, Ibiebele I, Liu C, Kopp A, Guttmann A, Ford J, van der Meulen J, Hjern A, Gilbert R. Preterm birth, unplanned hospital contact, and mortality in infants born to teenage mothers in five countries: An administrative data cohort study. Paediatr Perinat Epidemiol. (2020) 34(6):645-654. doi: 10.1111/ppe.12685
15. Hansen A, Moloney ME, Li J, Chavan NR. Preterm birth prevention in Appalachia: restructuring prenatal care to address rural health disparities and establish perinatal health equity. Health Equity. (2021) 21;5(1):203-209. doi: 10.1089/heq.2020.0064
16. Nguyen TT, Merchant JS, Criss S, Makres K, Gowda KN, Mane H, Yue X, Hswen Y, Glymour MM, Nguyen QC, Allen AM. Examining Twitter-derived negative racial sentiment as indicators of cultural racism: Observational associations with preterm birth and low birth weight among a multiracial sample of mothers, 2011-2021. J Med Internet Res. (2023) 25:e44990. doi: 10.2196/44990
17. Deriba BS. Nutritional-related predictors of preterm birth in North Shewa hospitals, central Ethiopia: A Case-Control Study. Pediatric Health Med Ther. (2021) 12:315-324. doi: 10.2147/PHMT.S319867

Environment

18. Coffman VR, Søndergaard Jensen A, Trabjerg BB, Pedersen CB, Hansen B, Sigsgaard T, et al. Prenatal exposure to nitrate from drinking water and the risk of preterm birth: A Danish nationwide cohort study. Environ Epidemiol. (2022) 6(5):e223. doi: 10.1097/EE9.0000000000000223
19. Ha S, Mendola P. Invited commentary: Ambient environment and the risk of preterm birth. Am J Epidemiol. (2017) 185(4):259-261. doi: 10.1093/aje/kww138.
20. Ncube CN, Enquobahrie DA, Albert SM, Herrick AL, Burke JG. Association of neighborhood context with offspring risk of preterm birth and low birthweight: A systematic review and meta-analysis of population-based studies. Soc Sci Med. (2016) 153:156-64. doi: 10.1016/j.socscimed.2016.02.014
21. Giurgescu C, Zenk SN, Dancy BL, Park CG, Dieber W, Block R. Relationships among neighborhood environment, racial discrimination, psychological distress, and preterm birth in African American women. J Obstet Gynecol Neonatal Nurs. (2012) 41(6):E51-61. doi: 10.1111/j.1552-6909.2012.01409.x.
22. Sun Y, Ilango SD, Schwarz L, Wang Q, Chen JC, Lawrence JM, et al. Examining the joint effects of heatwaves, air pollution, and green space on the risk of preterm birth in California. Environ Res Lett. (2020) 15(10):104099. doi: 10.1088/1748-9326/abb8a3
23. Mendola P. Invited commentary: The power of preterm birth to motivate a cleaner environment. Am J Epidemiol. (2018) 187(8):1595-1597. doi: 10.1093/aje/kwy108
24. Ferguson KK, Rosen EM, Barrett ES, Nguyen RHN, Bush N, McElrath TF, et al. Joint impact of phthalate exposure and stressful life events in pregnancy on preterm birth. Environ Int. (2019) 133(Pt B):105254. doi: 10.1016/j.envint.2019.105254

25. Ferguson KK, Chin HB. Environmental chemicals and preterm birth: Biological mechanisms and the state of the science. Curr Epidemiol Rep. (2017) 4(1):56-71. doi: 10.1007/s40471-017-0099-7

Food

26. Richterman A, Raymonville M, Hossain A, Millien C, Joseph JP, Jerome G, et al. Food insecurity as a risk factor for preterm birth: a prospective facility-based cohort study in rural Haiti. BMJ Glob Health. (2020) 5(7):e002341. doi: 10.1136/bmjgh-2020-002341
27. Madzia J, McKinney D, Kelly E, DeFranco E. Influence of Gestational Weight Gain on the Risk of preterm birth for underweight women living in food deserts. Am J Perinatol. (2021) 38(S 01):e77-e83. doi: 10.1055/s-0040-1705168
28. Robinson DT, Van Horn L, Balmert L, Silver RM, Parry S, Haas DM, et al. Dietary fat and fatty acid intake in nulliparous women: Associations with preterm birth and distinctions by maternal BMI. Curr Dev Nutr. (2021) 5(6):nzab074. doi: 10.1093/cdn/nzab074
29. Mikkelsen TB, Osterdal ML, Knudsen VK, Haugen M, Meltzer HM, Bakketeig L, et al. Association between a Mediterranean-type diet and risk of preterm birth among Danish women: a prospective cohort study. Acta Obstet Gynecol Scand. (2008) 87(3):325-30. doi: 10.1080/00016340801899347
30. Martin CL, Sotres-Alvarez D, Siega-Riz AM. Maternal dietary patterns during the second trimester are associated with preterm birth. J Nutr. (2015) 145(8):1857-64. doi: 10.3945/jn.115.212019

Education

31. Thoma ME, Drew LB, Hirai AH, Kim TY, Fenelon A, Shenassa ED. Black-White disparities in preterm birth: Geographic, social, and health determinants. Am J Prev Med. (2019) 57(5):675-686. doi: 10.1016/j.amepre.2019.07.007
32. Blumenshine PM, Egerter SA, Libet ML, Braveman PA. Father's education: an independent marker of risk for preterm birth. Matern Child Health J. (2011) 15(1):60-7. doi: 10.1007/s10995-009-0559-x.
33. Odd D, Evans D, Emond A. Preterm birth, age at school entry and long term educational achievement. PLoS One. (2016) 11(5):e0155157. doi: 10.1371/journal.pone.0155157
34. Antony KM, Levison J, Suter MA, Raine S, Chiudzu G, Phiri H, et al. Qualitative assessment of knowledge transfer regarding preterm birth in Malawi following the implementation of targeted health messages over 3 years. Int J Womens Health. (2019) 11:75-95. doi: 10.2147/IJWH.S185199

Work

35. Rodríguez-Fernández A, Ruíz-De la Fuente M, Sanhueza-Riquelme X, Parra-Flores J, Dolores Marrodán M, Maury-Sintjago E. Association between maternal factors, preterm birth, and low birth weight of Chilean singletons. Children (Basel). (2022) 9(7):967. doi: 10.3390/children9070967

36. Snelgrove JW, Murphy KE. Preterm birth and social inequality: assessing the effects of material and psychosocial disadvantage in a UK birth cohort. Acta Obstet Gynecol Scand. (2015) 94(7):766-775. doi: 10.1111/aogs.12648
37. Vrijkotte T, Brand T, Bonsel G. First trimester employment, working conditions and preterm birth: a prospective population-based cohort study. Occup Environ Med. (2021) 78(9):654-660. doi: 10.1136/oemed-2020-107072
38. Simpson JL. Are physical activity and employment related to preterm birth and low birth weight? Am J Obstet Gynecol. (1993) 168(4):1231-8. doi: 10.1016/0002-9378(93)90374-r.
39. Saurel-Cubizolles MJ, Zeitlin J, Lelong N, Papiernik E, Di Renzo GC, Bréart G. Europop Group. Employment, working conditions, and preterm birth: results from the Europop case-control survey. J Epidemiol Community Health. (2004) 58(5):395-401. doi: 10.1136/jech.2003.008029
40. Wheeler SM, Massengale KEC, Adewumi K, Fitzgerald TA, Dombeck CB, Swezey T, et al. Pregnancy vs. paycheck: a qualitative study of patient's experience with employment during pregnancy at high risk for preterm birth. BMC Pregnancy Childbirth. (2020) 20(1):565. doi: 10.1186/s12884-020-03246-7
41. Berkowitz GS, Papiernik E. Working conditions, maternity legislation, and preterm birth. Semin Perinatol. (1995) 19(4):272-8. doi: 10.1016/s0146-0005(05)80041-2

Water

42. Sherris AR, Baiocchi M, Fendorf S, Luby SP, Yang W, Shaw GM. Nitrate in drinking water during pregnancy and spontaneous preterm birth: A retrospective within-mother analysis in California. Environ Health Perspect. (2021) 129(5):57001. doi: 10.1289/EHP8205
43. Lewis C, Suffet IH, Hoggatt K, Ritz B. Estimated effects of disinfection by-products on preterm birth in a population served by a single water utility. Environ Health Perspect. (2007) 115(2):290-5. doi: 10.1289/ehp.9394
44. Brink LT, Nel DG, Hall DR, Odendaal HJ. Association of socioeconomic status and clinical and demographic conditions with the prevalence of preterm birth. Int J Gynaecol Obstet. (2020) 149(3):359-369. doi: 10.1002/ijgo.13143
45. Nyadanu SD, Tessema GA, Mullins B, Pereira G. Prenatal acute thermophysiological stress and spontaneous preterm birth in Western Australia, 2000-2015: A space-time-stratified case-crossover analysis. Int J Hyg Environ Health. (2022) 245:114029. doi: 10.1016/j.ijheh.2022.114029
46. Honest H, Forbes CA, Durée KH, Norman G, Duffy SB, Tsourapas A, et al. Screening to prevent spontaneous preterm birth: systematic reviews of accuracy and effectiveness literature with economic modelling. Health Technol Assess. (2009) 13(43):1-627. doi: 10.3310/hta13430
47. Stan CM, Boulvain M, Pfister R, Hirsbrunner-Almagbaly P. Hydration for treatment of preterm labour. Cochrane Database Syst Rev. (2013) 11:CD003096. doi:10.1002/14651858.CD003096.pub2

Health care

48. Ghosh C, Wojtowycz M. Effect of gestational disorders on preterm birth, low birthweight, and NICU admission. Arch Gynecol Obstet. (2021) 303(2):419-426. doi: 10.1007/s00404-020-05760-7
49. Siegel E. Low birth weight and preterm birth: the emerging importance of prevention. Soz Praventiv Med. (1985) 30(3):118-24. doi: 10.1007/BF02083156.
50. Jin W, Hughes K, Sim S, Shemer S, Sheehan P. The contemporary value of dedicated preterm birth clinics for high-risk singleton pregnancies: 15-year outcomes from a leading maternal centre. J Perinat Med. (2021) 49(9):1048-1057. doi: 10.1515/jpm-2021-0020
51. de Andrade L, Kozhumam AS, Rocha TAH, de Almeida DG, da Silva NC, de Souza Queiroz RC, et al. Impact of socioeconomic factors and health determinants on preterm birth in Brazil: a register-based study. BMC Pregnancy Childbirth. (2022) 22(1):872. doi: 10.1186/s12884-022-05201-0

Social support

52. Cross-Barnet C, Benatar S, Courtot B, Hill I, Johnston E, Cheeks M. Inequality and innovation: barriers and facilitators to 17P administration to prevent preterm birth among Medicaid participants. Matern Child Health J. (2018) 22(11):1607-1616. doi: 10.1007/s10995-018-2556-4
53. Kozhimannil KB, Hardeman RR, Alarid-Escudero F, Vogelsang CA, Blauer-Peterson C, Howell EA. Modeling the cost-effectiveness of doula care associated with reductions in preterm birth and cesarean delivery. Birth. (2016) 43(1):20-7. doi: 10.1111/birt.12218
54. Victora CG, Rubens CE. GAPPS Review Group. Global report on preterm birth and stillbirth (4 of 7): delivery of interventions. BMC Pregnancy Childbirth. (2010) 10 Suppl 1(Suppl 1):S4. doi: 10.1186/1471-2393-10-S1-S4
55. McLemore MR, Altman MR, Cooper N, Williams S, Rand L, Franck L. Health care experiences of pregnant, birthing and postnatal women of color at risk for preterm birth. Soc Sci Med. (2018) 201:127-135. doi: 10.1016/j.socscimed.2018.02.013
56. Pusdekar YV, Patel AB, Kurhe KG, Bhargav SR, Thorsten V, Garces A, et al. Rates and risk factors for preterm birth and low birthweight in the global network sites in six low- and low middle-income countries. Reprod Health. (2020) 17(Suppl 3):187. doi: 10.1186/s12978-020-01029-z
57. Carey JC, Klebanoff MA. National Institute of Child Health and Human Development Maternal-Fetal Medicine Units Network. What have we learned about vaginal infections and preterm birth? Semin Perinatol. (2003) 27(3):212-6. doi: 10.1016/s0146-0005(03)00020-x
58. Hetherington E, Doktorchik C, Premji SS, McDonald SW, Tough SC, Sauve RS. Preterm birth and social support during pregnancy: a systematic review and meta-analysis. Paediatr Perinat Epidemiol. (2015) 29(6):523-35. doi: 10.1111/ppe.12225
59. Surkan PJ, Dong L, Ji Y, Hong X, Ji H, Kimmel M, et al. Paternal involvement and support and risk of preterm birth: findings from the Boston birth cohort. J Psychosom Obstet Gynaecol. (2019) 40(1):48-56. doi: 10.1080/0167482X.2017.1398725

Housing

60. Sealy-Jefferson S, Butler B, Chettri S, Elmi H, Stevens A, Bosah C, et al. Neighborhood evictions, marital/cohabiting status, and preterm birth among African American women. Ethn Dis. (2021) 31(2):197-204. doi: 10.18865/ed.31.2.197
61. Krieger N, Van Wye G, Huynh M, Waterman PD, Maduro G, Li W, et al. Structural racism, historical redlining, and risk of preterm birth in New York City, 2013-2017. Am J Public Health. (2020) 110(7):1046-1053. doi: 10.2105/AJPH.2020.305656
62. Berhie SH, Riley LE, Bryant AS. Disparities in 17-Hydroxyprogesterone Caproate Offer and Uptake in Pregnancies at a Risk of Preterm Birth. Am J Perinatol. (2019) 36(10):1066-1071. doi: 10.1055/s-0038-1676113
63. Leung JY, Leung GM, Schooling CM. Socioeconomic disparities in preterm birth and birth weight in a non-Western developed setting: evidence from Hong Kong's 'Children of 1997' birth cohort. J Epidemiol Community Health. (2016) 70(11):1074-1081. doi: 10.1136/jech-2015-206668
64. Batra K, Pharr J, Olawepo JO, Cruz P. Understanding the multidimensional trajectory of psychosocial maternal risk factors causing preterm birth: A systematic review. Asian J Psychiatr. (2020) 54:102436. doi: 10.1016/j.ajp.2020.102436
65. Giurgescu C, Misra DP, Slaughter-Acey JC, Gillespie SL, Nowak AL, Dove-Medows E, et al. Neighborhoods, racism, stress, and preterm birth among African American women: A review. West J Nurs Res. (2022) 44(1):101-110. doi: 10.1177/01939459211041165
